# Pyloric Mucosal Diaphragm Associated with Hypertrophic Pyloric Stenosis: An Unusual Combination in a Male Neonate

**DOI:** 10.1055/s-0040-1713809

**Published:** 2020-08-07

**Authors:** Eugenie Chariot, Anna Poupalou, Julie Estievenart, Martine Dassonville, Erwin Vanderveken, Henri Steyaert

**Affiliations:** 1 Department of Pediatric Surgery, Université Libre de Bruxelles (ULB), HUDERF Hospital (Hôpital Universitaire des Enfants Reine Fabiola), Brussels, Belgium; 2 Department of Pediatric Surgery, Centre Hospitalier de Jolimont, La Louviere, Belgium

**Keywords:** hypertrophic, pyloric, diaphragm, stenosis, congenital

## Abstract

We describe herein the case of a 3-week-old baby with persistent nonbilious vomiting, due to a hypertrophic pyloric stenosis (HPS) associated with a congenital pyloric mucosal diaphragm. So far, an association between the two conditions has not been described. The diagnosis of a pyloric mucosal diaphragm was delayed because of its cooccurrence with HPS.

## Introduction

Gastric outlet obstruction in the first months of life, most of the time, is due to hypertrophic pyloric stenosis. 1 It is a clinical syndrome presenting with nonbilious postprandial vomiting in newborns. It occurs in 1.5 to 3 of 1,000 births. Among them, several other rare conditions may also be responsible for gastric outlet obstruction, 2 including a diaphragm or web. Mucosal diaphragms are congenital abnormalities that are part of the atresias of the upper digestive tract. 3 A diaphragm is a typical type-1 atresia and can either be duodenal, antral (most of the time) or pyloric. 4 The latter is the rarest and has only been described in a few case reports. 5 6 Herein, we present the case of a child who presented with hypertrophic pyloric stenosis and was found to have an additional pyloric web. The two conditions are not known to be associated.

## Case Presentation

A 3-week-old male was born at full term after a normal pregnancy. After a normal first 10 days of life, he started to present nonbilious postprandial simple vomiting. He was admitted to the pediatric department of a peripheral hospital because of repeated episodes of vomiting. After a few days, vomiting turned into projectile vomiting.

A sonography was performed and showed a pylorus with a channel length of 17 mm and a mural thickening to 4 mm, confirming the diagnosis of hypertrophic pyloric stenosis, and he was transferred to the pediatric surgical department.

An extramucosal pyloromyotomy (Bianchi's approach) was performed the day after, at 20 days of life. In the postoperative course, vomiting persisted, and a control sonography suspected the persistence of a stenosis since it showed no passage through the pylorus during the 8 minutes of the exam. This led the surgeons to a redo pyloromyotomy 5 days later by extending the incision a few millimeters more distally.

**Fig. 1:**
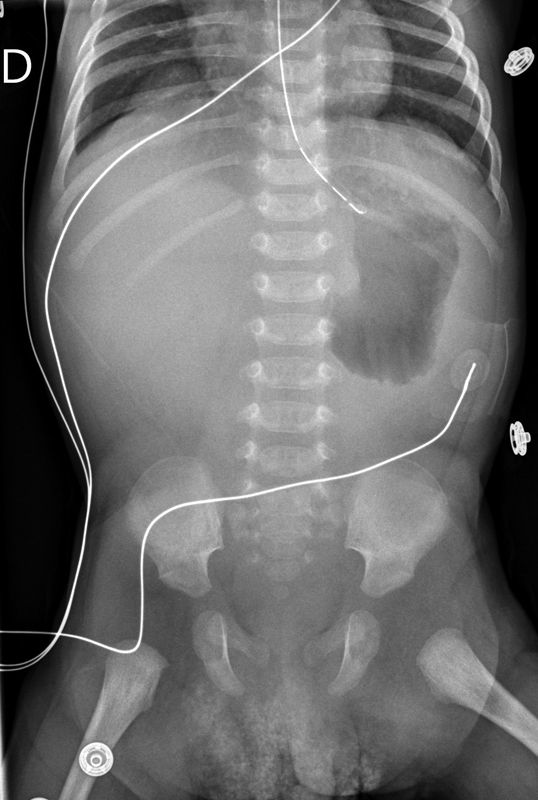
Dilated stomach at 5 weeks of age.

**Fig. 2:**
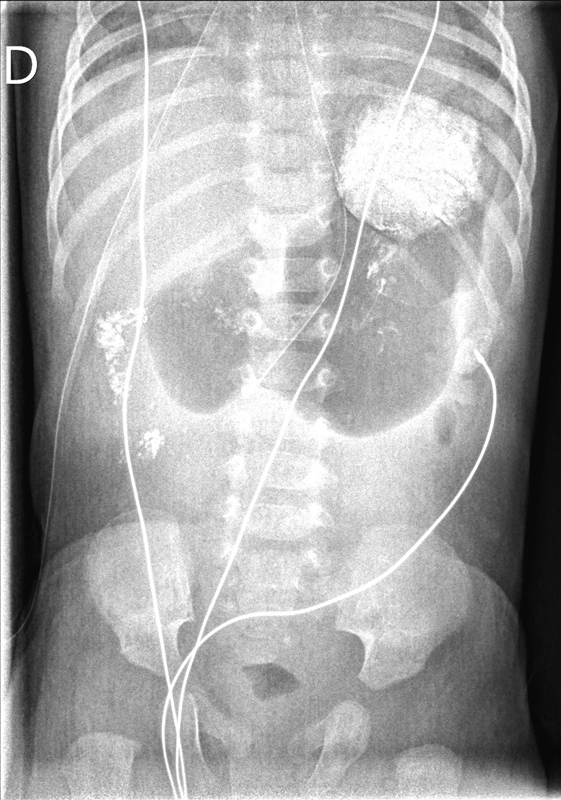
Dilated stomach and minimal passage of the contrast to the duodenum at 5 weeks of age.

Again, the vomiting didn't stop after the second surgery. The baby was on parenteral nutrition and his clinical condition was good. A radiography and an upper gastrointestinal (GI) study were performed at 5 weeks of age, and showed a stagnation of contrast in the dilated stomach and minimal passage to the duodenum ( Figs. 1 and 2 ).

The newborn was transferred to our third level reference children's hospital, for a second advice and to provide further care. An antral diaphragm was suspected as a differential diagnosis of gastric outlet obstruction. Our pediatric gastroenterologists refused to perform a gastroscopy, as they were concerned of the high risks of injuring the pylorus or creating a wrong way because of the recent surgeries.

**Fig. 3:**
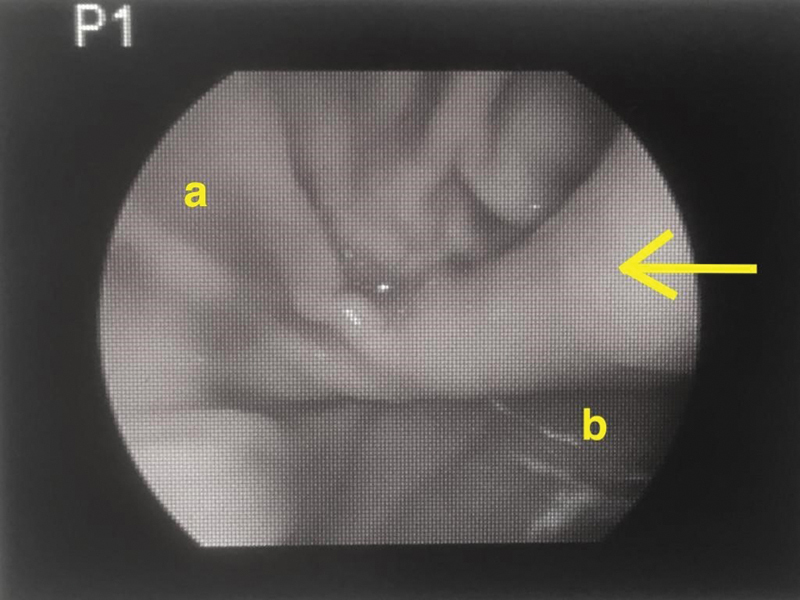
Laparoscopic view of the mucosal diaphragm (arrow). a, proximal part (stomach); b, distal part (duodenum).

The 5.5-week-old boy was finally taken back to the operating room (OR) for an open explorative laparotomy, during which a single 5-mm trocar was inserted into the fundus to insufflate the stomach and be able to look for an eventual endoluminal obstruction. With the help of this peroperative “laparoscopic” approach, we identified a mucosal fold within the pylorus ( Fig. 3 ). The pylorus was completely opened and the mucosal fold that obstructed the lumen was resected.

The postoperative course was uneventful. Nutrition was started at day 2 postoperatively through a jejunal tube left in place during the operation and gradually increased (while parenteral nutrition was gradually decreased). He was transferred in the peripheral hospital on day 5 postoperatively and discharged on day 14 postoperatively. He remains well 14 months after his discharge.

## Discussion

In this article, we report the case of a congenital pyloric diaphragm associated with hypertrophic pyloric stenosis. Congenital pyloric atresia (CPA) is a very rare congenital surgical abnormality, first described by Calder in 1733. 7

Its incidence is approximately 1 in 100,000 live births. CPA may occur as an isolated condition or associated with other congenital anomalies, in particular, junctional epidermolysis bullosa and hereditary multiple intestinal atresias. 8 Neonates with CPA should be screened for other abnormalities of the esophagus, genitourinary tract, heart, and skeleton.

CPA is divided anatomically in the following three types: (1) pyloric membrane or web type 1 (57%), (2) pyloric atresia without gap (pyloric tissue replaced by solid cord) type 2 (34%), and (3) pyloric atresia with a gap between stomach and duodenum type 3 (9%). 3 The obstruction may be complete or incomplete.

Nonbilious vomiting soon after birth without abdominal distention is the typical presentation of CPA. Pyloric atresia or complete web is usually accompanied by maternal polyhydramnios, epigastric distention, and early onset of the intestinal obstruction with “projectile” vomiting. Incomplete obstruction or incomplete web may appear later in the life, the onset is mild and the diagnosis may be missed or delayed. The recommended surgical treatment depends on the anatomical type of the anomaly. Membrane excision and Heineke–Mikulicz pyloroplasty is usually advocated in CPA type 1, excision of the atretic segment and gastroduodenostomy in type 2, and gastroduodenostomy in type 3. 3 8 9 The endoscopic approach to manage this unusual disease has been reported. It is indicated mostly in older children. High level of experience and suitable equipment is required. 10

Intraoperatively, it is important to exclude the presence of multiple diaphragms or atresias.

With the laparoscopic view of the antrum and pylorus, we could exclude the presence of another diaphragm. More distal combined atresia or diaphragm can be excluded by a meticulous examination of the bowel during surgery but also with the insertion of a gastric tube into the lumen and injection of saline to objectivate any obstacle. To our knowledge, the association between a congenital pyloric diaphragm and a hypertrophic pyloric stenosis has not been described in the literature so far. Only a few cases of antral diaphragms associated to hypertrophic pyloric stenosis have been reported. 11 12

## Conclusion

The combination of those two pathologies is exceptional but must be known to avoid, in some cases, unnecessary reoperations. When a newborn, diagnosed with HPS, starts vomiting again after an extramucosal pyloromyotomy, surgeons should keep in mind the possibility of an associated pyloric diaphragm, even more when HPS is diagnosed outside the typical age group (between 3 and 10 weeks). An upper GI contrast study should be scheduled, and if contrast study shows no or minimal passage of contrast through the pylorus and a redo operation is scheduled, the authors suggest that a gastroscopy should be performed intraoperatively to exclude this rare condition. If a simultaneous gastroscopy is performed intraoperatively, a possible injury to the pylorus can be detected and repaired.

## Notes

New Insights and the Importance for the Pediatric Surgeon A significant lesson to learn: when postprandial vomiting persists after a pyloromyotomy for hypertrophic pyloric stenosis, its association with the rare condition of congenital pyloric diaphragm should be kept in mind.
